# Sex differences in the aging murine urinary bladder and influence on the tumor immune microenvironment of a carcinogen-induced model of bladder cancer

**DOI:** 10.1186/s13293-022-00428-0

**Published:** 2022-05-03

**Authors:** Ali Hamade, Deyang Li, Kathrin Tyryshkin, Minqi Xu, Gwenaelle Conseil, Priyanka Yolmo, Jake Hamilton, Stephen Chenard, D. Robert Siemens, Madhuri Koti

**Affiliations:** 1 Queen’s Cancer Research Institute, Kingston, ON Canada; 2 Department of Biomedical and Molecular Sciences, Queen’s University, Kingston, ON Canada; 3 Department of Pathology and Molecular Medicine, Queen’s University, Kingston, ON Canada; 4 Department of Urology, Queen’s University, Kingston, ON Canada; 5 Department of Obstetrics and Gynecology, Queen’s University, Kingston, ON K7L3N6 Canada

**Keywords:** Bladder cancer, Tumor immune microenvironment, B cells, Tertiary lymphoid structure, BBN carcinogen, Sex differences

## Abstract

Sex and age associated differences in the tumor immune microenvironment of non-muscle invasive bladder (NMIBC) cancer and associated clinical outcomes are emerging indicators of treatment outcomes. The incidence of urothelial carcinoma of the bladder is four times higher in males than females; however, females tend to present with a more aggressive disease, a poorer response to immunotherapy and suffer worse clinical outcomes. Recent findings have demonstrated sex differences in the tumor immune microenvironment of non-muscle invasive and muscle invasive bladder cancer and associated clinical outcomes. However, a significant gap in knowledge remains with respect to the current pre-clinical modeling approaches to more precisely recapitulate these differences towards improved therapeutic design. Given the similarities in mucosal immune physiology between humans and mice, we evaluated the sex and age-related immune alterations in healthy murine bladders. Bulk-RNA sequencing and multiplex immunofluorescence-based spatial immune profiling of healthy murine bladders from male and female mice of age groups spanning young to old showed a highly altered immune landscape that exhibited sex and age associated differences, particularly in the context of B cell mediated responses. Spatial profiling of healthy bladders, using markers specific to macrophages, T cells, B cells, activated dendritic cells, high endothelial venules, myeloid cells and the PD-L1 immune checkpoint showed sex and age associated differences. Bladders from healthy older female mice also showed a higher presence of tertiary lymphoid structures (TLSs) compared to both young female and male equivalents. Spatial immune profiling of *N*-butyl-*N*-(4-hydroxybutyl) nitrosamine (BBN) carcinogen exposed male and female bladders from young and old mice revealed a similar frequency of TLS formation, sex differences in the bladder immune microenvironment and, age associated differences in latency of tumor induction. These findings support the incorporation of sex and age as factors in pre-clinical modeling of bladder cancer and will potentially advance the field of immunotherapeutic drug development to improve clinical outcomes.

**Supplementary Information:**

The online version contains supplementary material available at 10.1186/s13293-022-00428-0.

Bladder cancer accounts for 200,000 annual deaths globally [1] and presents as non-muscle invasive bladder cancer (NMIBC) or muscle invasive bladder cancer (MIBC). Bladder cancer remains the most management intensive and expensive cancer to treat in the elderly. Because of the high mutational burden, bladder tumors exhibit high immunogenicity. Patients with intermediate or high-risk NMIBC receive intravesical therapy with an attenuated strain of *Mycobacterium bovis* (*M. bovis*) also known as bacillus Calmette–Guérin (BCG; commonly used as a vaccine for prevention of tuberculosis) [2]. Despite the use of BCG immunotherapy as a gold standard for over 45 years, up to 50% patients exhibit poor responses, with many suffering from recurrence and progression to MIBC. Additionally, several other immunomodulatory therapies including adenoviral gene delivery vector-based approaches have shown some promise in the BCG non-responsive patients.

With an average age of 73 years at diagnosis, bladder cancer exhibits four times higher incidence in males compared to females. Female patients with NMIBC generally present late  with more advanced tumor stages than males, and often suffer early recurrences [3–6]. Bladder cancer also exhibits significant sex disparity in outcomes following immunomodulatory treatments [3, 7, 8]. Several previous reports have shown the influence of hormones and sex chromosome-associated genetic links mediating such sex differences; however, the influence of immunological factors remains less studied. With biological aging, the immune system experiences significant decline in function. Interestingly, this phenomenon, also known as immunosenescence, progresses in a sex-differential manner [3, 9–12]. The bladder is an organ with a mucosal barrier providing protection from uropathogens and therefore exhibits unique immunological characteristics that sustain a local physiological microbiome [13–15]. Simultaneous to changes in peripheral immune profiles, significant alterations within the mucosae also occur with biological aging and were recently reported in the context of healthy aging female murine bladder [16].

While the significance of age and sex as factors associated with incidence and clinical outcomes in bladder cancer have been well acknowledged [3, 7, 8], current pre-clinical modeling approaches do not incorporate these variables in experimental design. Furthermore, although the widely used orthotopic cancer cell implantation based syngeneic murine models are useful in addressing the mechanistic interactions associated with response to targeted therapies, these may not fully reflect the events associated with local and systemic immune physiological shifts and functional alterations that accompany biological aging in a sex-differential manner during carcinogenesis. This is more critical in the context of BCG immunotherapy in the elderly, where this therapy also acts as an adjuvant for both innate and adaptive immune compartments. Importantly, due to the ease and consistency of catheterization procedures, female mice are generally used in most of the implantation models of bladder cancer. Exposure to the carcinogen *N*-butyl-*N*-(4-hydroxybutyl) nitrosamine (BBN), a mutagen mimicking cigarette smoke, recapitulates the most common risk factor in bladder cancer [17]. This widely used bladder cancer model also exhibits tumor mutational spectrum and heterogeneity and has been employed for investigations in both NMIBC and MIBC [18, 19]. Several additional genetically engineered and transgenic models of bladder cancer have been developed; however, the age and sex-associated local immune physiological alterations in the urinary bladder have yet to be fully characterized.

In the current study, we first characterized the age and sex associated whole transcriptome profiles, with a focus on immune response pathways, in the urinary bladders of physiologically healthy female and male mice. We then characterized sex differences in the pre-treatment bladder immune microenvironment of the BBN carcinogen-induced syngeneic murine model of NMIBC across various age groups. Findings from this study provide a comprehensive overview of the immunological changes that accompany aging processes in female and male murine urinary bladders and emphasize the need to incorporate age and sex as factors in pre-clinical modelling of bladder cancer. 

## Materials and methods

All murine experimental procedures were approved by the Queen’s University Animal Care Committee. Wild type C57BL6/J male and female mice belonging to age groups ranging from young to old [20] were  purchased  from Charles River Laboratory (Montreal, QC, Canada). Mice were fed a sterilized conventional diet and water and maintained under specific  pathogen-free environment.

### Bulk-RNA sequencing and gene set enrichment analysis

Whole bladders were collected from 3-, 6-, 9-, 12-, 15- and 18-month-old healthy female and male mice (*n* = 3–4 each age group and sex) and snap frozen in liquid nitrogen. Simultaneously, whole bladders were formalin fixed for histological evaluation (*n* = 5–8 in each sex for each age group) using hematoxylin and eosin (H&E) staining. These age groups were selected to mimic the young (3–6 months), middle (9–12) and older (15–18) ages in mice, as previously defined [20]. Total RNA from fresh-frozen whole bladders was isolated using the RNeasy Mini Kit (Qiagen Inc, Canada). Qualitative and quantitative assessments of RNA were performed using a spectrophotometer (Nanodrop ND-100) and a bioanalyzer (Agilent). Samples that met the purity requirement (> 8.0 RNA integrity number) were subjected to bulk RNA-sequencing (Genome Quebec, Montreal, QC, Canada). The RNA-Seq raw and processed data files have been deposited to NCBI Gene Expression Omnibus (GEO accession GSE191087 at http://www.ncbi.nlm.nih.gov/projects/geo/). RNA-sequencing data analysis was performed using previously established protocols [21]. Pre-ranked lists resulting from DESeq2 analysis were subjected to Gene Set Enrichment Analysis (GSEA; http://software.broadinstitute.org/gsea/) for comparison against GO Biological Processes gene sets for a preliminary gene ontology analysis (version 7.4). Findings were validated using a proprietary feature selection algorithm (Tyryshkin K., unpublished) after transcripts per million (TPM) normalized counts were filtered to isolate genes with the top 10% of expression to find the top 1% of differentially expressed genes between comparison groups. Top 200 differentially expressed genes (identified via DESeq2 analysis) from each comparison within and between sexes were also subjected to further analysis to identify enrichment of genes predicting cell subsets using the MyGeneset application (https://www.immgen.org/) [22, 23]. Details of additional analysis are provided in Additional file 6: Additional Methods.

### BBN carcinogen treatment

Female and male C57BL/6J mice belonging to young (5 months), middle (12 months) and older (15 months) age groups (*n* = 15–20 each age group) were administered 0.05% *N*-butyl-*N*-(4-hydroxybutyl) nitrosamine (BBN; TCI America) ad libitum in drinking water (protected from light) once a week for 12 weeks as per previously established protocols [24]. Ultrasound-based imaging was performed at 4-week intervals to monitor changes in the bladder wall. Bladders were collected at 4, 7 and 12 weeks (*n* = 5 at each time point) post-initiation of BBN exposure to characterize the bladder immune microenvironment by histopathology and multiplex immunofluorescence (IF).

### Multiplex immunofluorescence staining

Following histological evaluation using H&E staining at the Queen’s Molecular Pathology Laboratory, paraffin-embedded tissue sections (4 μm) were subjected to spatial immune profiling using multiplex IF staining to analyze infiltration patterns of selected immune markers using two panels of antibodies to identify CD11b+ myeloid cells, CD3+ total T cells, CD8+ cytotoxic T cells, PNAd+ high endothelial venules, Pax5+ B cells, PD-L1 immune check point, CD163+ M2-like macrophages, EpCAM+ epithelial cells (Additional file 2: Table S1). IF staining was performed at the Molecular and Cellular Immunology Core (MCIC) facility, BC Cancer Agency as per previously reported methods [8]. Multiplex IF stained images were acquired using the Vectra 3 multichannel imaging system. Images were then imported to Phenochart (1.1.0) and Inform Viewer (2.5.0) software for annotation followed by capturing at high magnification. The Inform Viewer (version 2.5.0, Akoya Biosciences) was used to visualize a detailed composite image of each section with the flexibility of spectral separation of all the fluorophores from each panel. StarDist (https://github.com/stardist/stardist), a deep learning tool extension available in the QuPath software (https://qupath.github.io) was used to further analyze the expression of individual markers within a region of interest (ROI) [25]. Positive staining thresholds were manually defined for each marker following confirmation using the DAPI nuclear stain channel. Immune cell infiltration was evaluated in 3–11 random high-power fields/ROIs adjacent to the basal layer of the urothelium in the bladders from healthy and BBN-treated mice. Average number of cells calculated per mm [2] were exported to GraphPad Prism (version 9.0) to analyze differences between sexes and ages. In Prism, the mean of all ROIs (*n* = 3–11) per mouse was used for subsequent analysis. Normalcy was assessed with the Shapiro–Wilk test, which indicated that some counts had unequal variances between sexes. Thus, an unpaired Welch’s *t*-test was used to determine significant differences in cell densities between sexes.

## Results

### Bulk-RNA sequencing reveals age and sex-associated differences in transcriptome profiles of healthy murine urinary bladders

We characterized the local immune profiles of the healthy murine urinary bladders to determine whether any age-associated differences existed between females and male mice. To measure the age-related transcriptomic alterations, we first performed an unsupervised feature selection following the pre-processing of the RNA-seq files. Unsupervised heatmap clustering using the top 25% ranked genes automatically distinguished the female and male sexes in all age groups (representative comparisons from two age groups are shown in Additional file 1: Fig. S1). Significant differences in immune response genes within and between females and males across different age groups were observed (Additional file 3: Table S2, Additional file 4: Table S3 and Additional file 5: Table S4). These findings confirmed that sex differences in murine urinary bladder transcriptome are present across different ages and amplify with increasing age.

### B cell function-associated pathways are enriched in bladders from healthy aged females compared to aged males

**Fig. 1:**
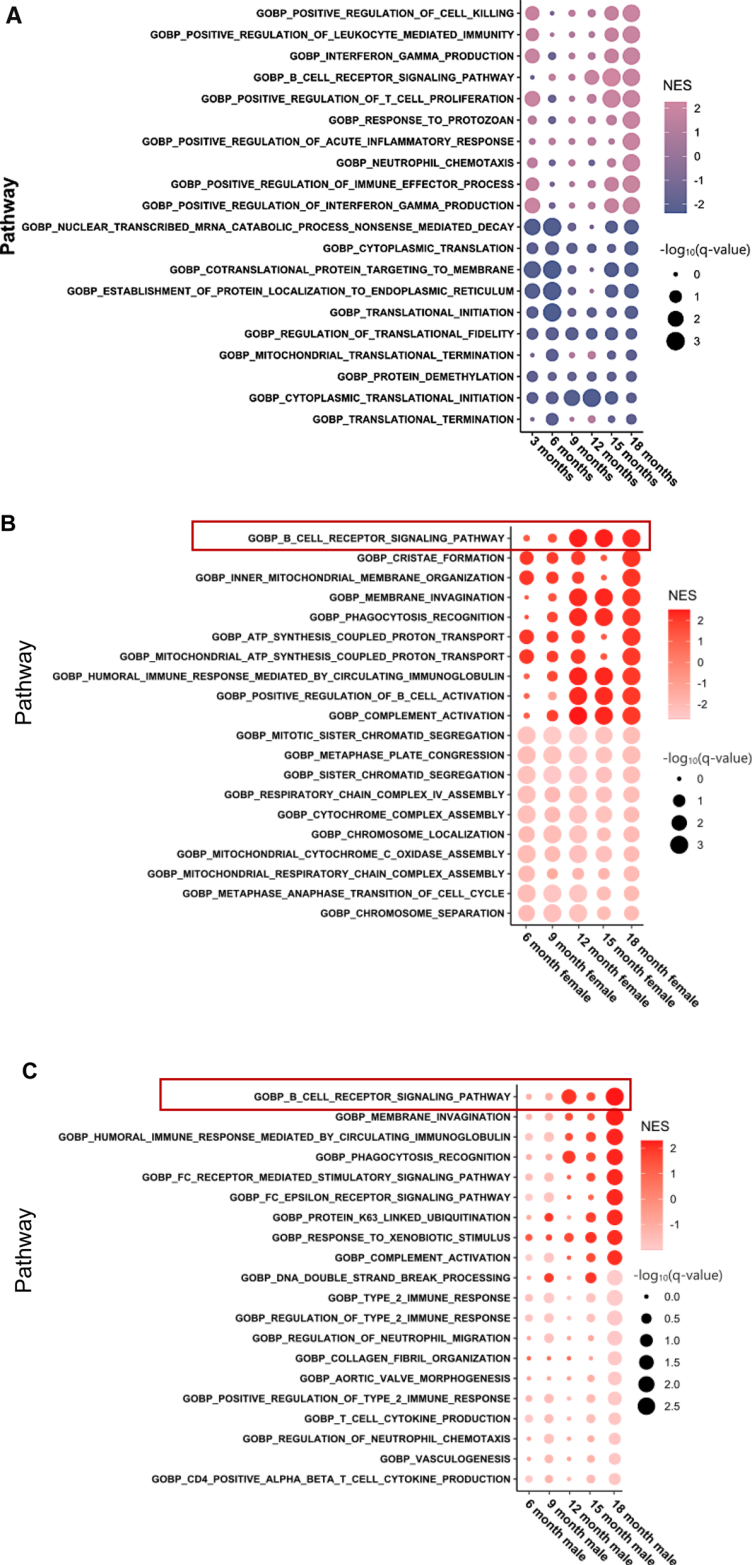
Urinary bladder of healthy C57BL/6 mice exhibit age and sex-associated differences in immune response pathways. Enrichment of the top 10 significantly enriched and downregulated GO biological processes pathways in **A** females compared to age-matched males, **B** aged females compared to 3-month-old females and, **C** aged males compared to 3-month-old males. Total RNA from healthy male and female urinary bladders from different age groups was subjected to bulk-RNA sequencing. Differential expression was assessed using DESeq2 in R (1.4.0). GSEA pre-ranked analysis of whole transcriptomic log2foldchange ranked lists from DESeq2 was performed. Plots were generated using ggplot2 (3.3.5), with dot size corresponding to −log10(*q*-value, FDR), and the color associated with normalized enrichment score (NES). Red box highlights the enrichment in B cell receptor signaling pathway

Pathway enrichment was performed with the gene set enrichment analysis (GSEA) tool using whole transcriptomic log2fold change ranked lists obtained from DESeq2 analysis (Additional file 3: Table S2, Additional file 4: Table S3 and Additional file 5: Table S4). GSEA analysis revealed enrichment in pathways associated with B cell receptor signaling and B cell-mediated immunity among the top 10 enriched pathways starting at 12 months of age (Fig. 1A–C). While similar enrichment was observed upon comparison of the 18-month-old group with the 3-month-old group within both sexes, between sex comparisons revealed significant increase in enrichment score in 18-month-old female compared to their male counterparts (Fig. 1A). The 12-, 15-, and 18-month-old females had a significantly greater enrichment (*q*-value < 0.05, false discovery rate; FDR) of B cell and other immune-related biological processes compared to 3-month-old female mice (Fig. 1B). Cytoscape visualization of all significantly upregulated GO pathways within and between sexes in the aged vs. young context (*q*-value < 0.05, FDR), further confirmed that multiple pathways associated with immune response and B-cell function cluster together with similar age associated trends within and between sexes (Additional file 1: Fig. S2).

**Fig. 2:**
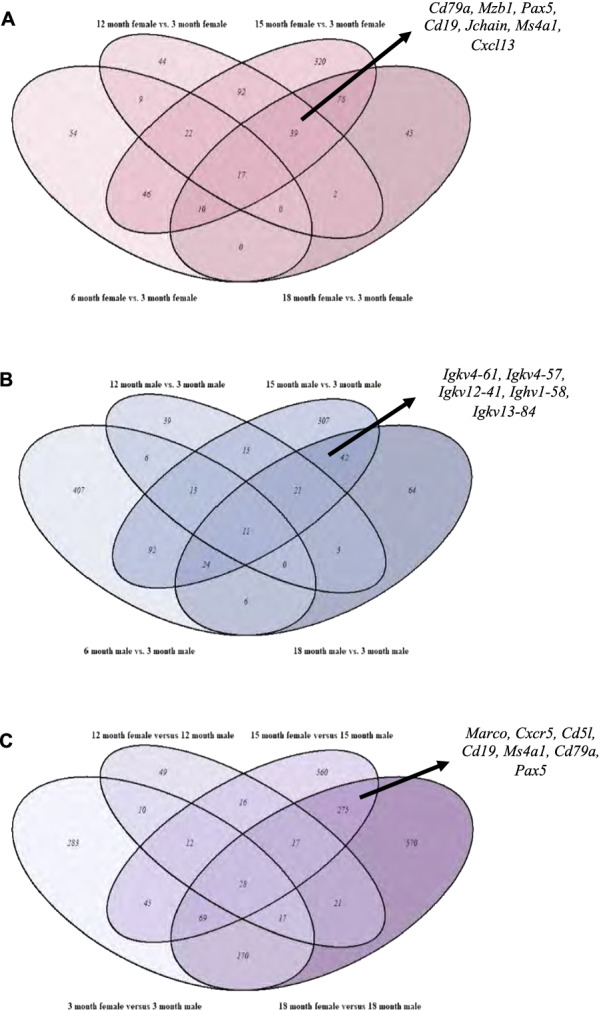
Sex differences in transcriptomic profiles of aging murine urinary bladders. Venn diagrams showing age and sex associated overlaps and intersecting transcriptomic profiles of significantly differentially upregulated genes in aged mice compared to 3-month-old mice within **A** females **B** males, and between **C** age-matched males and females. Examples of genes associated with B cells are indicated by arrows. Total RNA from healthy male and female urinary bladders was subjected to bulk-RNA sequencing. Differential expression was assessed using DESeq2 program in R (1.4.0) with a *P*_adj_ < 0.05 (FDR) and a log2fold change cut off of 0.58. Venn diagrams were generated from resulting gene lists using the VennDiagram (1.6.2) package in R

**Fig. 3:**
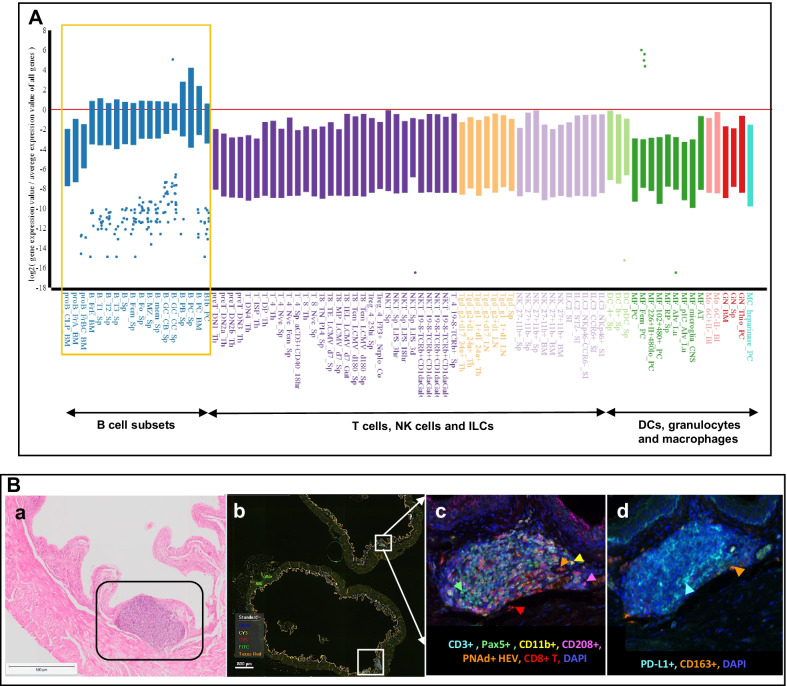
Age associated increase in B cell populations in female mouse bladder. **A** Healthy male and female mouse bladders (*n* = 3/4) of ages, 3, 6, 9, 12, 15 and 18 months were subjected to bulk RNA-Seq. Differential expression was assessed using DESeq2 in R (1.4.0) with a *P-*adj < 0.05 (FDR). Analysis of differential expression gene lists was performed using the MyGeneset application at https://www.immgen.org/, to determine immune cell subset type enrichment. Plot represents comparison between top 200 differentially expressed genes between 15-month-old female and age matched males. **B** Hematoxylin and eosin stained section showing lymphoid aggregate in an 18-month-old female mouse bladder wall (**a**). Whole bladder image captured using Phenochart Software image viewer 1.0.9 (Akoya Biosciences) from multiplex immunofluorescence stained 18-month-old female bladder section showing tertiary lymphoid structures (**b**; white boxes). Immune cells within TLSs (F4/80+ macrophages, CD163+ M2 macrophages, Pax5+ B cells, CD3+ T cells, CD8+ cytotoxic T cells and PD-L1 immune checkpoint (**c**, **d**). Representative of *n* = 3–5 in each sex

As illustrated by the Venn diagram of gene lists significantly upregulated in 6-, 12-, 15- and 18-month-old females compared to the 3-month-old females (log2fold change cutoff of 0.58, adjusted P value (*P*-adj) < 0.05), B cell-associated genes (*Cxcr5*, *Cxcl13*, *Cd19*, *Cd79a*, *Pax5*, *Mzb1*, *Ms4a1*; Additional file 3: Table S2 and Additional file 5: Table S4) were commonly differentially expressed in aged females (upregulated in 12-, 15-, 18-month females; Fig. 2). Similar analysis within males (Additional file 4: Table S3) and between males and females (Additional file 5: Table S4), revealed a comparable age-associated relationship, but between 15- and 18-month age groups only. Notably, these overlapping gene lists included a large number of immune response-associated genes in aged female mice compared to young mice or age-matched male mice. In addition, Immgen MyGeneset application-based immune cell prediction further showed increased shifts in various B cell subset-associated genes initiating at 12 months in females, whereas in males, similar increases were observed at 18 months of age. Between sex comparisons revealed significantly increased expression of genes associated with different B-cell populations in females starting at 15 months (Fig. 3A).

### Tertiary lymphoid structure formation occurs in the healthy bladder mucosa with biological aging

Histopathological evaluation followed by multiplex IF showed higher numbers of lymphoid aggregates/TLSs within the lamina propria of aged females (Fig. 3B; panel a). In both male and female mice, these aggregates first appeared at 9 months of age and with a larger diameter in female bladders. Multiplex IF staining revealed the presence of B cells, T cells, cytotoxic T, CD11b myeloid cells, and high-endothelial venule (HEV) confirming a TLS (Fig. 3B; panel c). Only the aged female groups appeared to have organized TLS structures within the lamina propria. Given the regulation of PD-L1 immune checkpoint by X-chromosome-associated miRNAs, we also measured its protein expression levels in this study. Higher expression of PD-L1 was observed within the TLSs along with higher infiltration of CD163+ cells in 18-month-old mice from both sexes (Fig. 3B; panel d). These findings are suggestive of sex differences in mechanisms underlying aging-associated increased magnitude of inflammatory changes within the urinary bladder.

### BBN carcinogen treatment leads to distinct age and sex-associated immunologic alterations in the bladder microenvironment

**Fig. 4:**
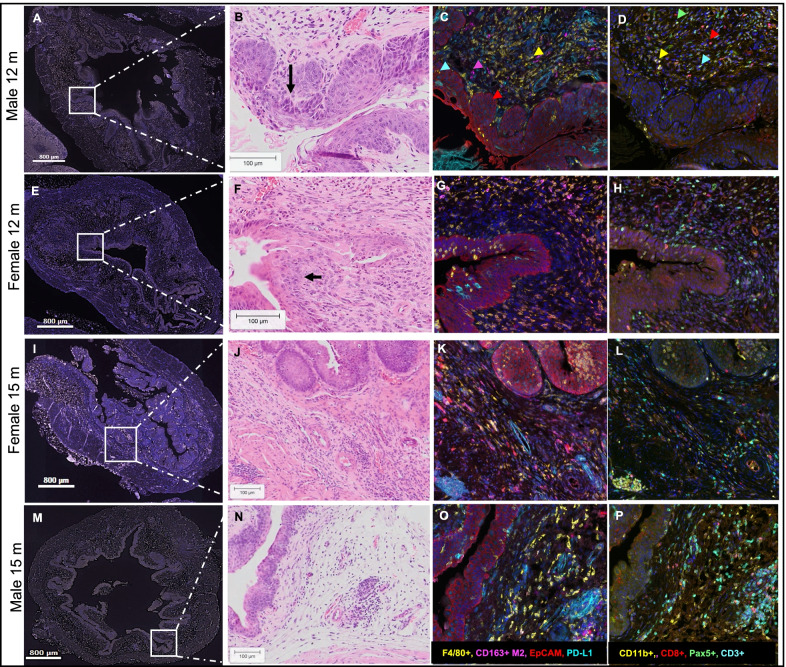
Bladder immune microenvironment following BBN carcinogen exposure in male and female mice. Whole bladder image captured using Phenochart Software image viewer 1.0.9 (Akoya Biosciences) showing low magnification image of multiplex immunofluorescence stained whole bladders from 12- and 15-month old male (**A**, **M**) and female (**E**, **I**), respectively, at 12 weeks post BBN exposure. Corresponding H&E stained section of a male (**B**, **N**) and female (**F**, **J**) bladder showing high-grade dysplasia, carcinoma in situ and early invasion. Multiplex immunofluorescence stained image showing F4/80+ macrophages, CD163+ M2-like macrophages, PD-L1 immune checkpoint, EpCAM+ epithelial cells (**C**, **G**, **K** and **O**) and CD11b+ myeloid cells, Pax5+ B cells, CD8+ cytotoxic T cells and CD3+ T cells (**D**, **H**, **L**, **P**). High magnification (×20) mages representative of *n* = 3–11 regions of interest from bladders of 3 to 5 mice in each sex

Based on the findings from healthy murine bladder transcriptomic profiling, we next sought to define the age group that more closely reflects the systemic and local immunologic functional states at the time of local treatment initiation in NMIBC. Female and male mice belonging to young, middle, and older age groups were exposed to BBN carcinogen for 12 weeks. Histopathological evaluation of bladders collected at various time points revealed the presence of reactive atypia at 4 weeks and the presence of urothelial dysplasia at 7 weeks post-BBN in the mice from the younger age group. Presence of dysplasia with focal carcinoma in situ was present at 12 weeks post-BBN in 100% of mice in this age group. In the 12-month-old male mice, early stages of invasion to lamina propria was present in 3 out of 5 mice at 12 weeks post-BBN administration (Fig. 4B). In age-matched female mice, invasion to lamina propria was observed in one out of 5 mice and the remaining 4 exhibited dysplasia with widespread carcinoma in situ at this time point (Fig. 4F). Interestingly, in the 15-month-old groups, invasion to lamina propria and formation of TLSs was present at 7 weeks post-BBN in both sexes. Intensity of hyperplasia was higher in both sexes in this age group (Fig. 4J, N). However, the abundance of plasma cells in the lamina propria of the female mice from the older age groups was more pronounced in comparison to young females and males from both age groups in the study (Additional file 1: Fig. S4C, D). Another important finding was the higher magnitude of overall immune cell infiltration in the lamina propria, accompanied with edematous changes, in both young and old female mice (Additional file 1: Fig. S4A–D). Thickening of the bladder wall due to these changes was also observed via ultrasound imaging (data not shown). Myeloid cells were the most abundant across all age groups. Although both F4/80+ and CD163+ macrophages were present at a high density, significant increase in only F4/80+ macrophages (Additional file 1: Fig. S6A) was observed in 12-month-old female bladders at 12 weeks post-BBN (Fig. 4G). Increased expression of PD-L1 on urothelium and endothelial lining of vessel walls was observed in the lamina propria of bladders at 12 weeks post-BBN (Fig. 4C). Most importantly and in line with local immune responses at mucosal surfaces, TLS formation as a result of carcinogen exposure was present in the lamina propria of all BBN carcinogen-treated mice in all age groups (Fig. 4L; Additional file 1: Fig. S5C, D). Overall, these findings indicate an influence of age on tumor progression in both sexes.

## Discussion

Biological aging-related sex-disparity in bladder-associated pathologies such as bladder cancer as well as healthy bladder mucosal tissue has recently gained significant attention. Despite discrepancies in the literature and a consensus on age and sex-associated hormonal, genetic and immunological deviations, the current pre-clinical models of bladder cancer have yet to fully integrate such factors in experimental design for development of immunotherapeutic agents.

It is known that females usually mount stronger adaptive immune responses to infectious and vaccine associated immune challenges, producing higher antibody titers and more robust innate immune responses. With the similarities in mucosal immune physiology between humans and mice, and the recently reported age-associated, antigenic stimuli-independent inflammation in female murine bladders [16], we conducted a comprehensive evaluation of the sex and age-associated bladder transcriptomic and spatial immune profiling in female and male mice. Not surprisingly, an unsupervised feature selection applied to detect differences in transcriptomic profiles of bladders from all age groups led to distinct clustering of groups by age and sex. While this confirmed the unique bladder transcriptome profiles of the groups under study, many of the top upregulated genes revealed shifts in immune-associated genes with increasing age, suggesting that fundamental changes in the aged murine bladder tissue microenvironment were immune-related. These findings are in concordance with the previous report by Ligon et al. [16], where such changes were reported only in female mice.

Significant enrichment in pathways associated with B cell function was observed in the bladder transcriptome profiles, starting at 9 months of age in both sexes. Evaluation of the top 25% significantly differentially expressed genes in the oldest group revealed increased expression of a substantial number of transcripts associated with a B cell phenotype such as *Cd19*, *Cd79a*, *Pax5*, *Mzb1*, *Ms4a1* and others. The age-associated increased B cell density was independently revealed by multiplex spatial immune profiling of the healthy bladder, prominently in aged female mice, that also exhibited an increased prevalence of TLSs. Following birth, such mucosa-associated lymphoid tissues (MALTs), consisting of T cells, B cells, macrophages and follicular dendritic cells, develop across various mucosal sites (e.g., respiratory tract, gut, urogenital tract). The neogenesis of TLSs occurs as a result of exposure to commensal or pathogenic microbes, IFN-1 activating vaccines that are administered via mucosal routes, or chronic local inflammation. Such induced TLSs generally provide heterologous humoral immune protection at mucosal sites [26]. As widely reported, TLSs in the bladder also form during the course of normal aging due to increased systemic levels of TNF-α [16], chronic inflammatory conditions of the urogenital tract such as those observed in interstitial cystitis-associated Hunner lesions and bladder cancer [27–30]. As per the recent TLS definitions distinguishing lymphoid aggregate, primary and secondary follicles [31], we did not observe a follicular dendritic cell network within TLSs of 18 months mouse bladders. Therefore, age-related bladder mucosal TLS formation is potentially induced by physiological increase in TNF-α with advancing age.

As observed through analysis using the Immgen MyGeneset application, the increased levels of transcripts associated with various B cell subsets are suggestive of multiple populations dispersed within the healthy bladder mucosa that may or may not be intrinsic to TLSs. Interestingly, the immunoglobulin light and heavy chain variable region genes constituted the majority of the top 50 genes distinguishing the profiles of aged female mice from the younger groups. Although such differences in these B cell-associated transcripts showed an increasing pattern of abundance with advancing age in both sexes, these observations were found to be more pronounced in bladders from 18-month-old female mice compared to all other groups.

In both humans and mice, B cells are known to exhibit age-related sex differences [12, 32]. With advancing immunologic and biologic aging, peripheral pools of B cells show significant decline with a simultaneous increase in mucosal surfaces. In the current study, the *Cxcr5* gene was also significantly overexpressed in the bladders of old females with concurrent higher expression of the gene *Cxcl13* that encodes the B cell-recruiting chemokine CXCL13. Higher transcripts levels of *J chain* also indicate a higher amount of secreted IgM and IgA antibodies in the bladder mucosa. Indeed, identification of the functional properties of these cells in the context of carcinogenesis and locally administered BCG immunotherapy in NMIBC is needed to augment the identification of newer therapeutic targets.

Another interesting and novel observation in our study was the increased expression of *Pd-l1* immune checkpoint gene in bladder transcriptome profiles of aged healthy female mice, which was further also observed at the protein level within the TLSs, reflective of exhausted immune cell states. Increased expression of other immune checkpoint genes such as *Lag3* and *Btla*, was also observed in the bladders from healthy older females compared to their male counterparts. A bulk sequencing approach does not allow the identification of specific cell types that exhibit such exhausted behavior and thus characterization of cell type specific lack of function via approaches such as single cell sequencing would allow for a comprehensive understanding of such alterations and development of precise therapeutic targeting approaches.

In a recent report, Degoricija et al., showed the higher expression of immune checkpoint molecules in tumors from young male mice at 20 weeks following exposure to BBN carcinogen [33]. Although this is one of the few reports that have characterized some local inflammatory changes accompanying carcinogens, this study was conducted in young male mice. Based on our observation of increased PD-L1 protein expression in the urothelial and endothelial cells, it can be speculated that this may result from BBN induced DNA damage and activation of cellular IFN pathways. Such increased PD-L1 expression on endothelial cells is known to cause inhibition of T cell activation [34]. Future mechanistic studies targeting this checkpoint are thus warranted to define its dynamic role in disease progression and potential immune exclusion.

Findings from our study showing higher density of plasma cells in the lamina propria of old mice, irrespective of sex, aligns with the increased recruitment of B cells to the bladder mucosa with advancing age that we observed in our findings from healthy bladder molecular profiling. It is plausible that chronic BBN exposure further amplifies the recruitment and differentiation of B cells to plasma cells. This is supported by the observation that increase in immune infiltration and lymphoid aggregate formation were observed as early as 4 weeks post-BBN initiation in the majority of mice in all age groups.

The biological and immunological aging-related similarities in humans and mice, emphasize the inclusion of aged mice in pre-clinical studies in bladder cancer. This is more important especially in studies investigating locally delivered BCG immunotherapy in NMIBC, to establish the role of resident and treatment induced or recruited immune cells that may exhibit age and sex dependent function in treatment response. Indeed, tumor associated B cells and bladder tumor-associated TLSs are gaining increased attention because of their association with response to BCG (*unpublished findings*) in NMIBC and positive outcomes following immune checkpoint blockade in MIBC [29, 35]. Future in-depth investigations into the interactions between these pre-existing TLSs and locally delivered immunotherapy are necessary. Finally, of the other immune cell types such as cytotoxic T cells and myeloid cells, we observed a high density of both F4/80+ and CD163+ M2-like macrophages in the bladder immune microenvironment of BBN treated mice.

The majority of studies in bladder cancer are conducted in 6- to 8-week-old mice. The influence of hormones on sex differences in time to tumor induction in the BBN model was established in a seminal study over 4 decades ago [36]. However, the impact of immunologic aging that accompanies hormonal alterations remains to be fully understood. Moreover, mice do not undergo menopause and significant decline in estrogen levels as seen in humans [37], and exhibit the phenomenon of reproductive senescence associated decline in ovarian function. It is thus challenging to model the cross-talks between hormones and immune alterations precisely. Moreover, the role of androgen and androgen receptors in mediating BBN carcinogen associated sex differences in mice is well established [38, 39].

Our study is indeed, not without limitations. Age associated increased B cell infiltrated TLSs within the bladder mucosa were first demonstrated by Ligon et al. [16], in female wild type and TNF-α knockout mice housed under specific pathogen free and germ-free conditions. It will be interesting and important to expand these findings to TNF-α deficient male mice given the significantly high levels in elderly males [40]. Future investigations could also utilize new technologies such as single cell sequencing in models that permit distinguishing hormonal and chromosomal influences. Indeed, the four-core genotype model [41, 42] permits exploration of such questions. Nonetheless, our results lay the foundation for such future studies to understand the biological mechanisms underlying sex differences in the aging urinary bladder to develop improved therapeutics that benefit both females and males.

## Conclusions

Our novel findings on age and sex-associated immune landscape changes in the healthy murine urinary bladder and their potential influence on the immune microenvironment of the BBN carcinogen-induced model of bladder cancer, suggest that these factors are critical in pre-clinical modeling of the disease. Use of older mice in development of immune potentiating therapies for bladder cancer treatment will advance the current understanding of local immune responses and facilitate more precise translation to humans. Further investigations in the context of novel immunotherapies are justified to understand the influence  of age and sex in  treatment response and overall outcomes.

## Supplementary Information

**Additional file 1: Figure S1.** Unsupervised log2 mean-centered heatmap of RNA sequencing data of the top 25% ranked genes between and within sexes. **Figure S2.** C57BL/6 mice show increased immune associated pathway enrichment in an age- and sex-dependant manner. **Figure S3.** Age and sex related shifts in B cells in healthy murine urinary bladders. **Figure S4.** Hematoxylin and eosin (H&E) stained section from 5-month-old female (**A**) and age matched male (**B**) bladder at 12 weeks post BBN exposure. Tertiary lymphoid structure (black boxes) in the lamina propria of 15-month-old female (**C**) and age matched male (**D**) bladders at 7 weeks post BBN. **Figure S5.** Bladder microenvironment following exposure to BBN carcinogen. **Figure S6.** Differences in infiltration patterns of select immune markers between 12-month-old male and female mice. **Figure S7.** Cell Detection using StarDist extension tool of QuPath software.

**Additional file 2: Table S1.** Antibodies used in multiplex immunofluorescence staining.

**Additional file 3: Table S2.** Differentially expressed genes (P-adj < 0.05, FDR) within different age groups spanning young to old in female mice.

**Additional file 4: Table S3.** Differentially expressed genes (P-adj < 0.05, FDR) within different age groups spanning young to old in male mice.

**Additional file 5: Table S4.** Differentially expressed genes (P-adj < 0.05, FDR) between male and female mice across different age groups spanning young to old.

**Additional file 6.** Detailed methods for RNA-Seq data analysis.

## Data Availability

All RNA-sequencing has been deposited to Gene Expression Omnibus (GEO accession GSE191087 at http://www.ncbi.nlm.nih.gov/projects/geo/).

## Abbreviations

BCG: Bacillus Calmette–Guérin
BBN: *N*-Butyl-*N*-(4-hydroxybutyl) nitrosamine
FDR: False discovery rate
GO: Gene ontology
GSEA: Gene set enrichment analysis
IFN: Interferon
NMIBC: Non-muscle invasive bladder cancer (NMIBC)
MIBC: Muscle invasive bladder cancer (MIBC)
PD-L1: Programmed death ligand 1
TLS: Tertiary lymphoid structure
TPM: Transcripts per million
