# Supplementary material for: Sex differences in the aging murine urinary bladder and influence on the tumor immune microenvironment of a carcinogen-induced model of bladder cancer
Source: Biol Sex Differ. 2022 May 3;13:19. doi: 10.1186/s13293-022-00428-0 (PMC9066862; doi:10.1186/s13293-022-00428-0)
Supplement: Supplementary file 1 — Additional file 1: Figure S1. Unsupervised log2 mean-centered heatmap of RNA sequencing data of the top 25% ranked genes between and within sexes. Figure S2. C57BL/6 mice show increased immune associated pathway enrichment in an age- and sex-dependant manner. Figure S3. Age and sex related shifts in B cells in healthy murine urinary bladders. Figure S4. Hematoxylin and eosin (H&E) stained section from 5-month-old female (A) and age matched male (B) bladder at 12 weeks post BBN exposure. Tertiary lymphoid structure (black boxes) in the lamina propria of 15-month-old female (C) and age matched male (D) bladders at 7 weeks post BBN. Figure S5. Bladder microenvironment following exposure to BBN carcinogen. Figure S6. Differences in infiltration patterns of select immune markers between 12-month-old male and female mice. Figure S7. Cell Detection using StarDist extension tool of QuPath software. [file 13293_2022_428_MOESM1_ESM.pptx]

## Slide 1
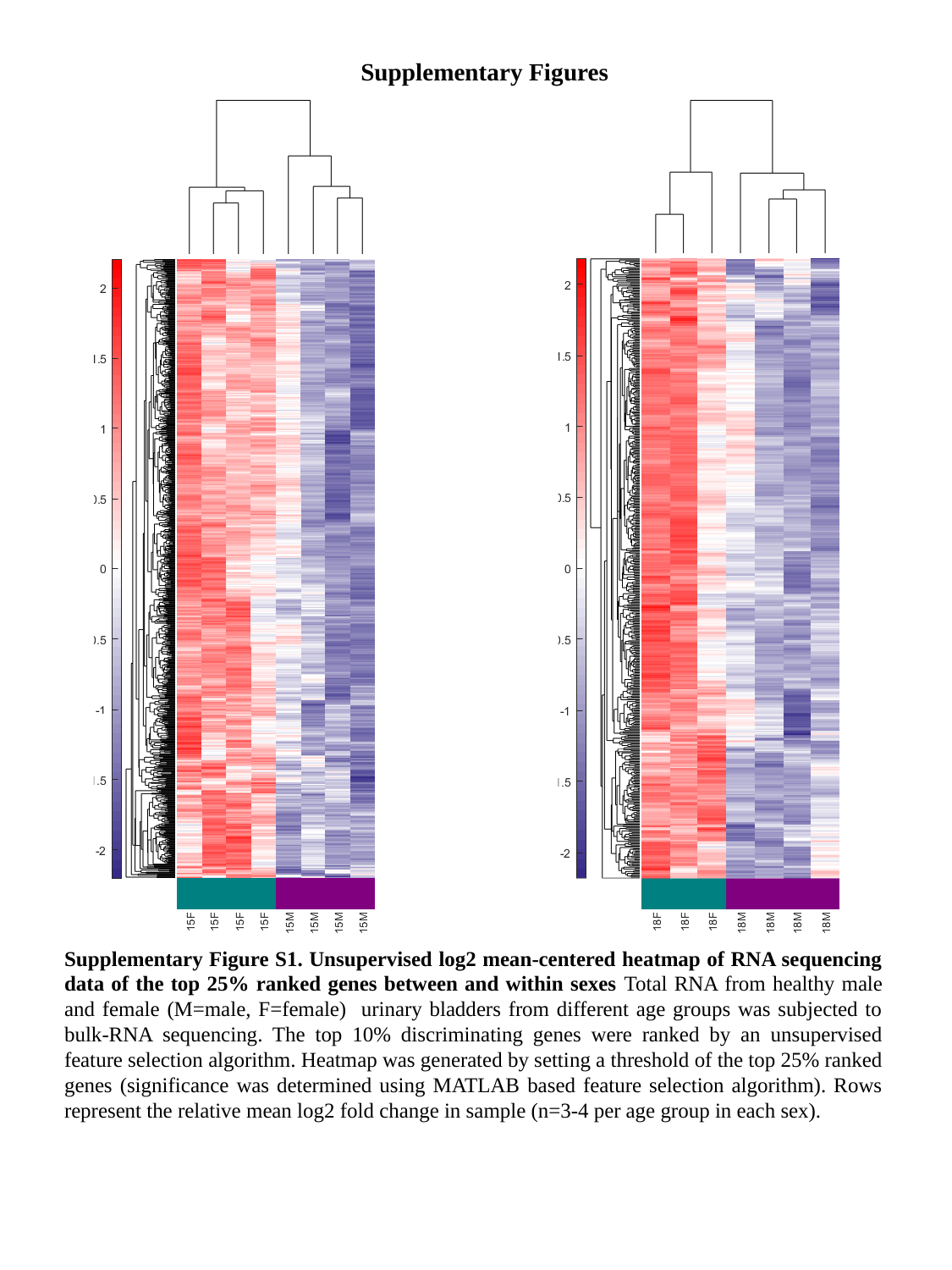

Supplementary Figures
Supplementary Figure S1. Unsupervised log2 mean-centered heatmap of RNA sequencing data of the top 25% ranked genes between and within sexes Total RNA from healthy male and female (M=male, F=female) urinary bladders from different age groups was subjected to bulk-RNA sequencing. The top 10% discriminating genes were ranked by an unsupervised feature selection algorithm. Heatmap was generated by setting a threshold of the top 25% ranked genes (significance was determined using MATLAB based feature selection algorithm). Rows represent the relative mean log2 fold change in sample (n=3-4 per age group in each sex).

## Slide 2
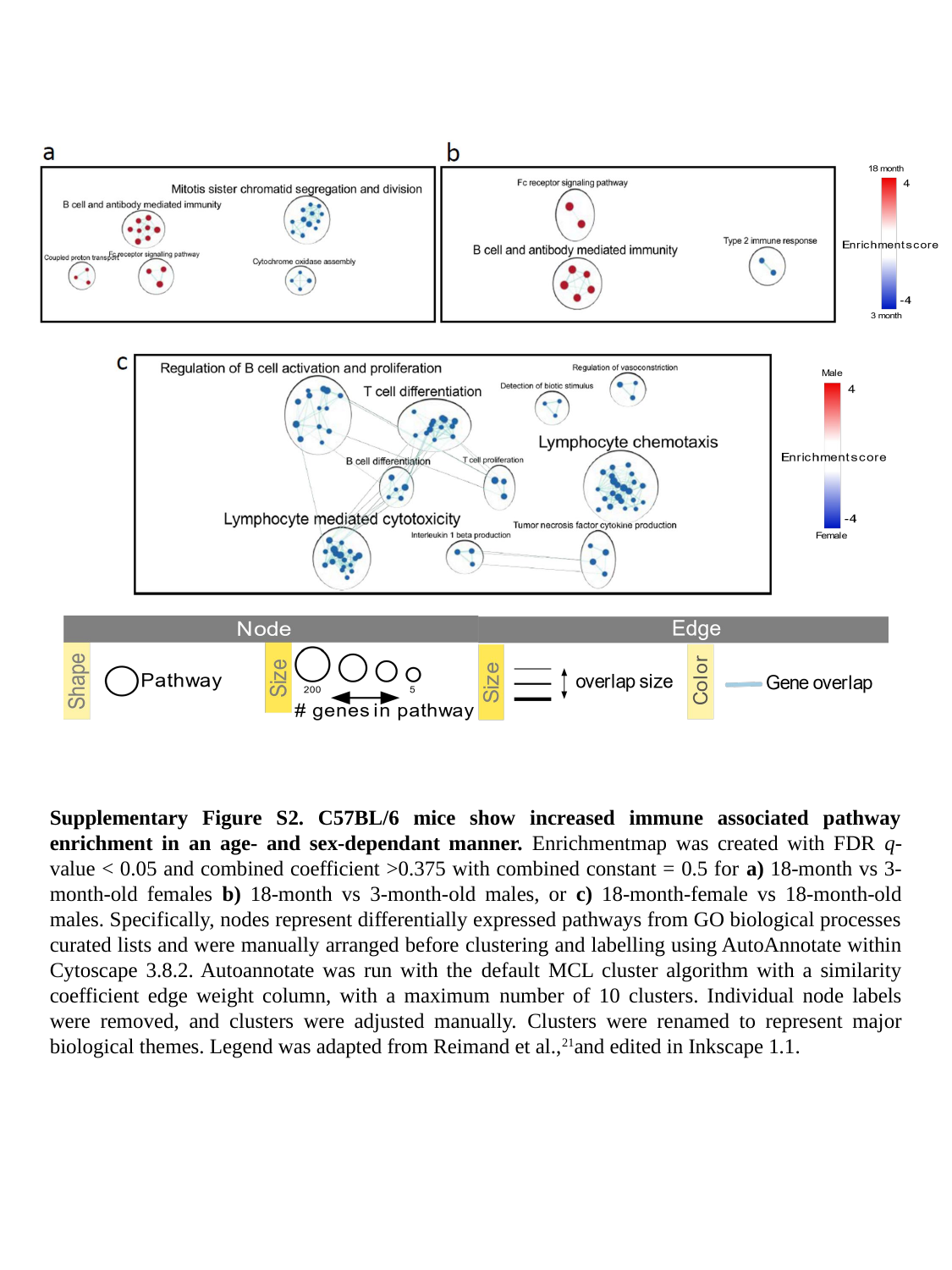

Supplementary Figure S2. C57BL/6 mice show increased immune associated pathway enrichment in an age- and sex-dependant manner. Enrichmentmap was created with FDR q-value < 0.05 and combined coefficient >0.375 with combined constant = 0.5 for a) 18-month vs 3-month-old females b) 18-month vs 3-month-old males, or c) 18-month-female vs 18-month-old males. Specifically, nodes represent differentially expressed pathways from GO biological processes curated lists and were manually arranged before clustering and labelling using AutoAnnotate within Cytoscape 3.8.2. Autoannotate was run with the default MCL cluster algorithm with a similarity coefficient edge weight column, with a maximum number of 10 clusters. Individual node labels were removed, and clusters were adjusted manually. Clusters were renamed to represent major biological themes. Legend was adapted from Reimand et al.,21and edited in Inkscape 1.1.

## Slide 3
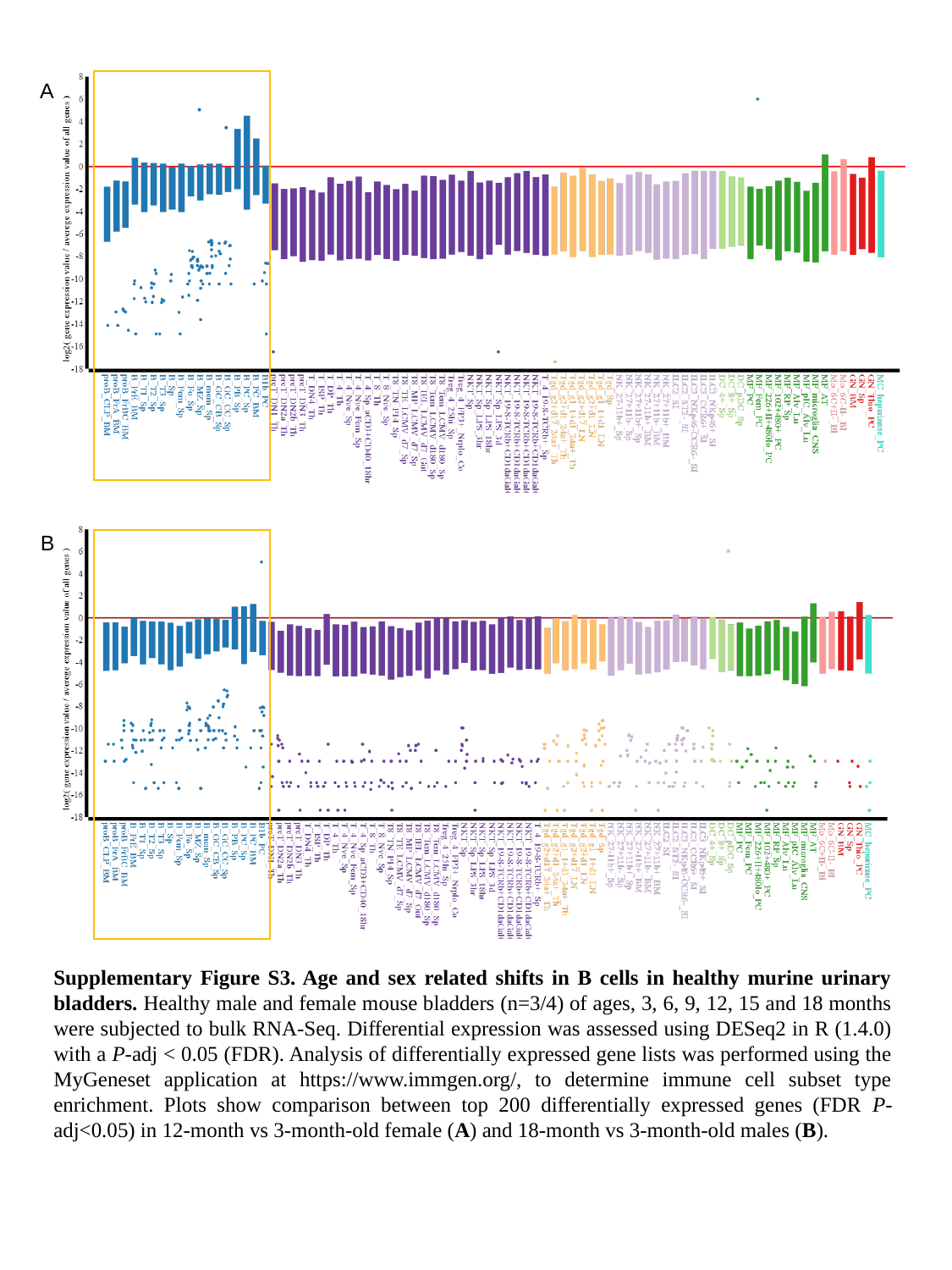

A
B
Supplementary Figure S3. Age and sex related shifts in B cells in healthy murine urinary bladders. Healthy male and female mouse bladders (n=3/4) of ages, 3, 6, 9, 12, 15 and 18 months were subjected to bulk RNA-Seq. Differential expression was assessed using DESeq2 in R (1.4.0) with a P-adj < 0.05 (FDR). Analysis of differentially expressed gene lists was performed using the MyGeneset application at https://www.immgen.org/, to determine immune cell subset type enrichment. Plots show comparison between top 200 differentially expressed genes (FDR P-adj<0.05) in 12-month vs 3-month-old female (A) and 18-month vs 3-month-old males (B).

## Slide 4
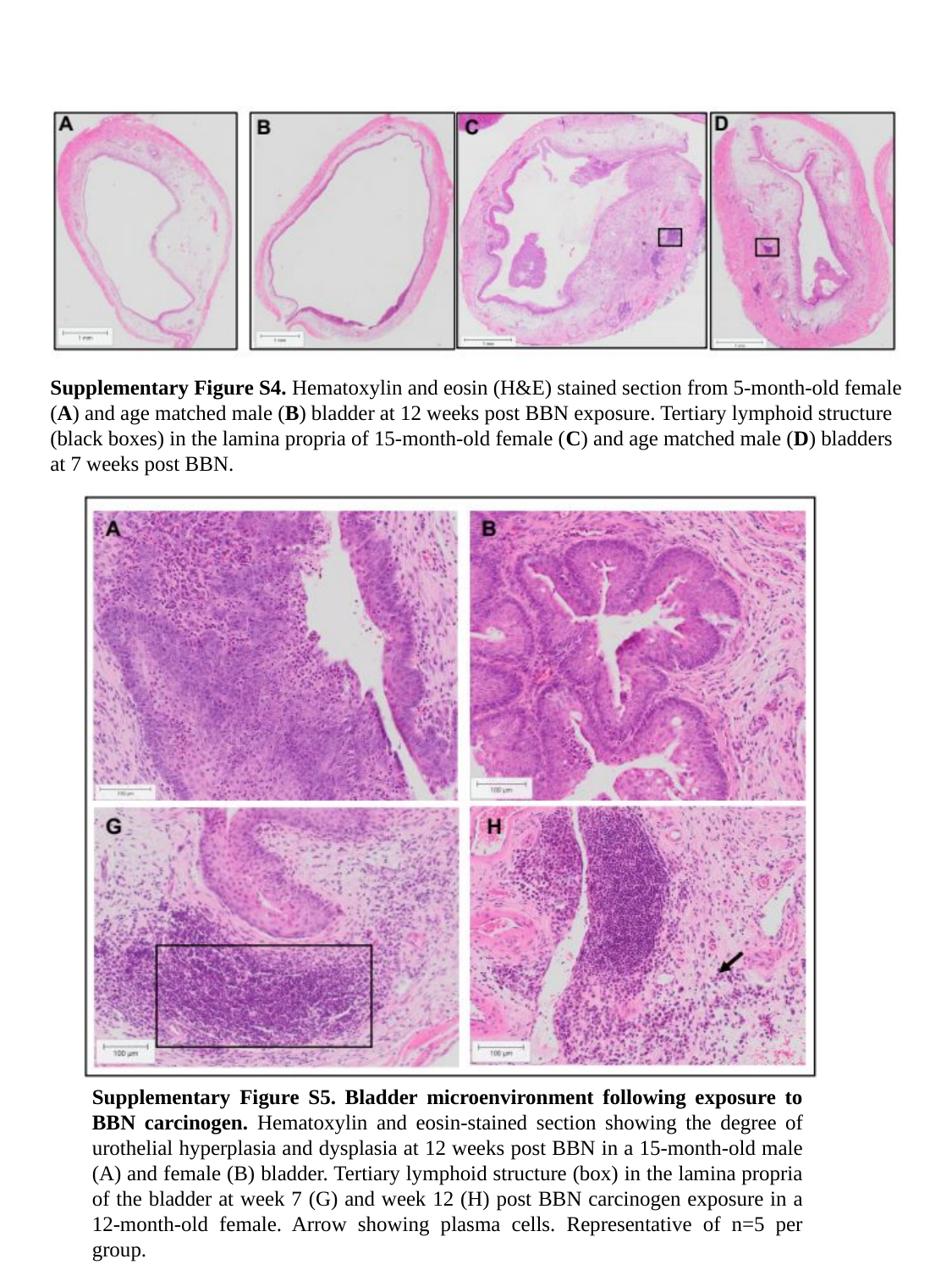

Supplementary Figure S4. Hematoxylin and eosin (H&E) stained section from 5-month-old female (A) and age matched male (B) bladder at 12 weeks post BBN exposure. Tertiary lymphoid structure (black boxes) in the lamina propria of 15-month-old female (C) and age matched male (D) bladders at 7 weeks post BBN.
Supplementary Figure S5. Bladder microenvironment following exposure to BBN carcinogen. Hematoxylin and eosin-stained section showing the degree of urothelial hyperplasia and dysplasia at 12 weeks post BBN in a 15-month-old male (A) and female (B) bladder. Tertiary lymphoid structure (box) in the lamina propria of the bladder at week 7 (G) and week 12 (H) post BBN carcinogen exposure in a 12-month-old female. Arrow showing plasma cells. Representative of n=5 per group.

## Slide 5
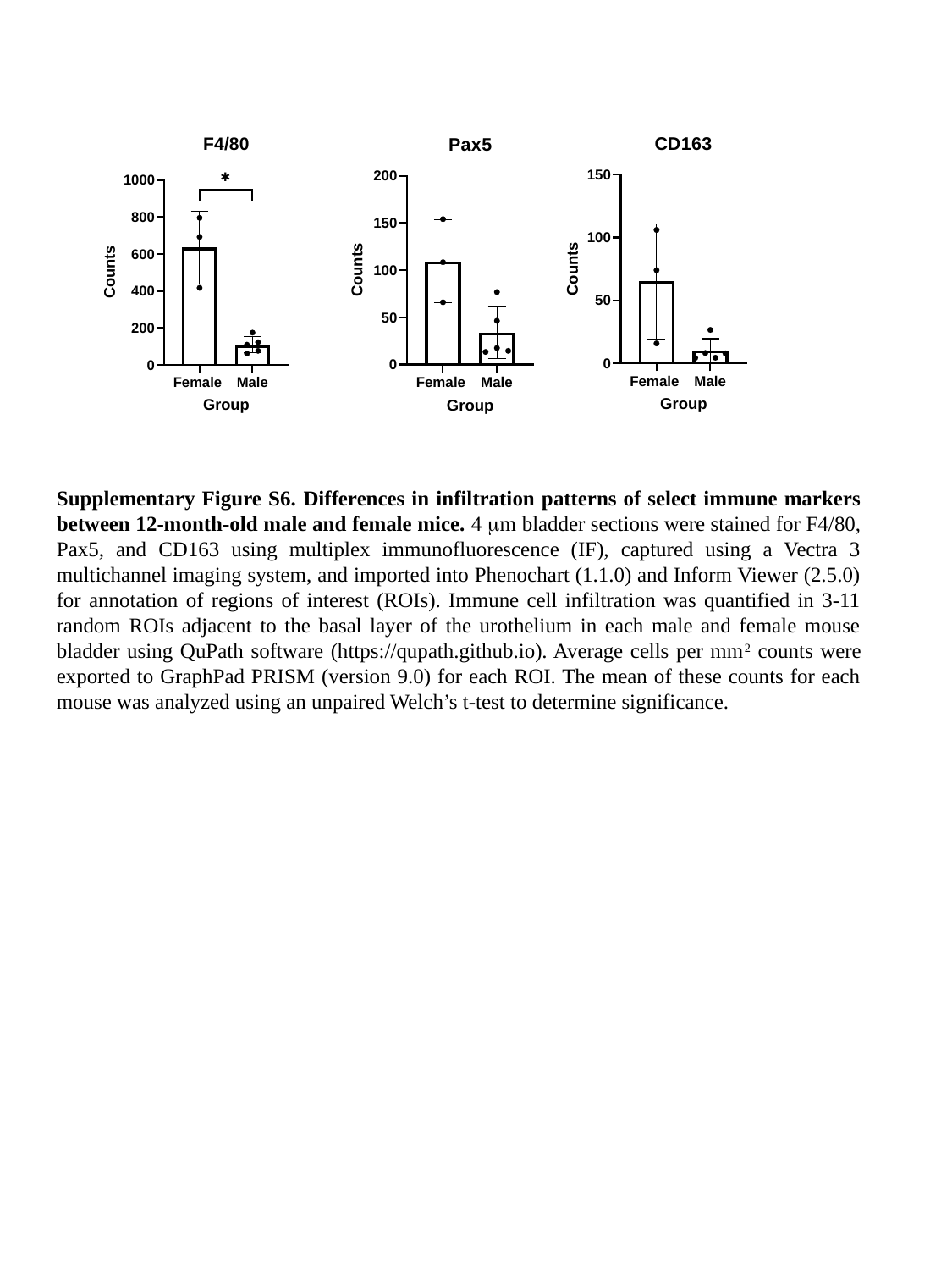

Supplementary Figure S6. Differences in infiltration patterns of select immune markers between 12-month-old male and female mice. 4 μm bladder sections were stained for F4/80, Pax5, and CD163 using multiplex immunofluorescence (IF), captured using a Vectra 3 multichannel imaging system, and imported into Phenochart (1.1.0) and Inform Viewer (2.5.0) for annotation of regions of interest (ROIs). Immune cell infiltration was quantified in 3-11 random ROIs adjacent to the basal layer of the urothelium in each male and female mouse bladder using QuPath software (https://qupath.github.io). Average cells per mm2 counts were exported to GraphPad PRISM (version 9.0) for each ROI. The mean of these counts for each mouse was analyzed using an unpaired Welch’s t-test to determine significance.

## Slide 6
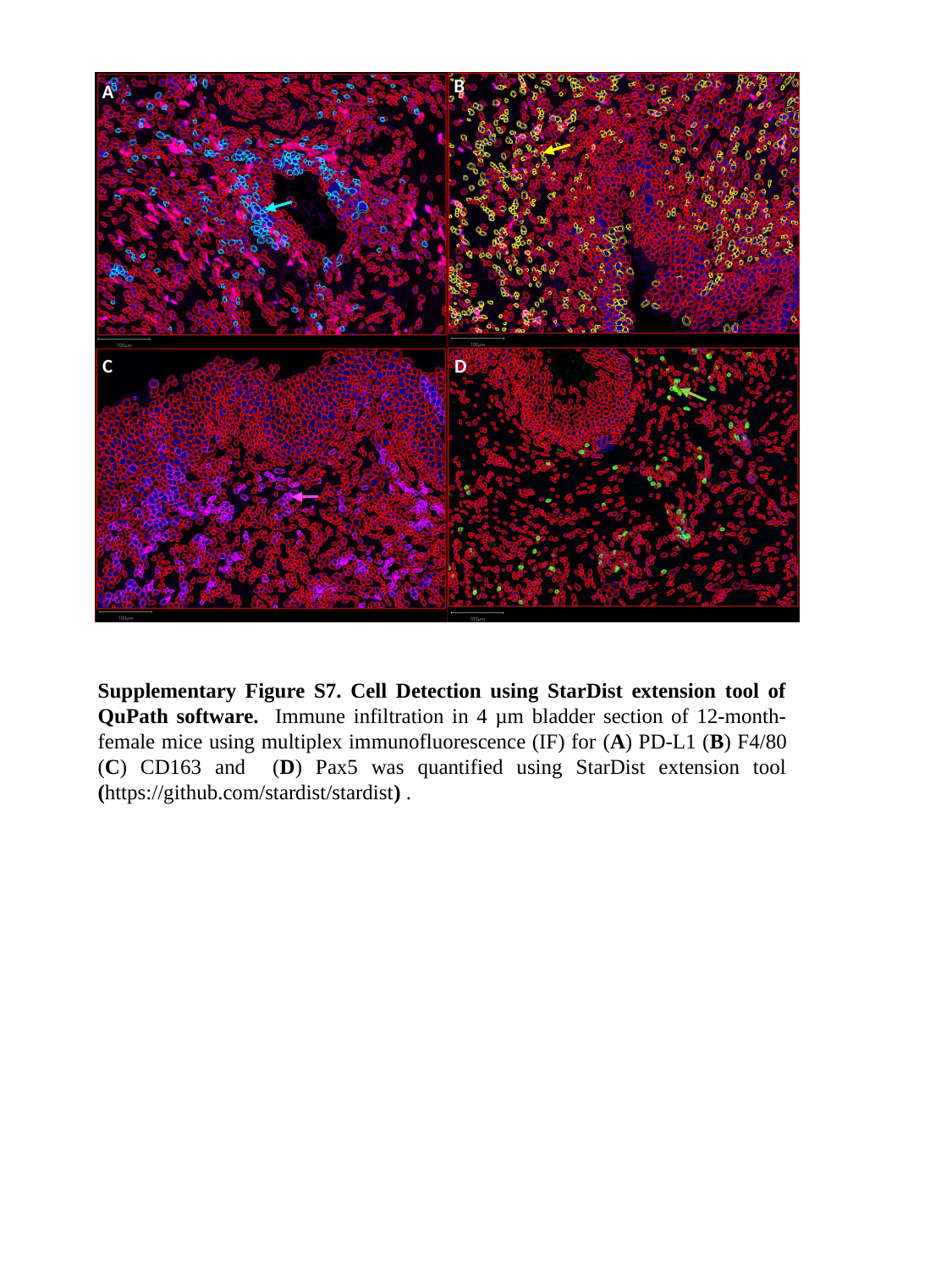

B
A
C
D
Supplementary Figure S7. Cell Detection using StarDist extension tool of QuPath software. Immune infiltration in 4 µm bladder section of 12-month-female mice using multiplex immunofluorescence (IF) for (A) PD-L1 (B) F4/80 (C) CD163 and (D) Pax5 was quantified using StarDist extension tool (https://github.com/stardist/stardist) .
