# Supplementary material for: Sex differences in the aging murine urinary bladder and influence on the tumor immune microenvironment of a carcinogen-induced model of bladder cancer
Source: Biol Sex Differ. 2022 May 3;13:19. doi: 10.1186/s13293-022-00428-0 (PMC9066862; doi:10.1186/s13293-022-00428-0)
Supplement: Supplementary file 2 — Additional file 2: Table S1. Antibodies used in multiplex immunofluorescence staining. [file 13293_2022_428_MOESM2_ESM.docx]

**Table S1. Antibodies used in multiplex immunofluorescence staining.**

| **Primary Antibody** | **Staining Round** | **Species** | **Cat#** | **Clone #** | **Ab Dilution (Diluent)** | **Fluorophore** | **Fluorophore dilution** |
| --- | --- | --- | --- | --- | --- | --- | --- |
| CD208 | 1 | Rat | DDX0191P-100 | 1010E1.01 | 1/100 RR | Opal 650 | 1/100 |
| CD3 | 2 | Rabbit | M3074 | SP7 | 1/600 DVG | Opal 520 | 1/300 |
| PNAd | 3 | Rat | 120802 | MECA-79 | 1/200 DVG | Opal 620 | 1/100 |
| Pax5 | 4 | Rabbit | ab109443 | EPR3730 | 1/1000 DVG | Opal 540 | 1/500 |
| CD8 | 5 | Rabbit | 98941S | D4W2Z | 1/200 DVG | Opal 690 | 1/150 |
| CD11b | 6 | Rabbit | ab133357 | EPR1344 | 1/7500 DVG | Opal 570 | 1/800 |
| CD163 | 1 | Rabbit | ab182422 | EPR19518 | 1/3000 RR | OPAL570 | 1/200 |
| Ly6G | 2 | Rat | 127607 | 1A8 | 1/800 DVG | OPAL620 | 1/300 |
| EpCAM | 3 | Rabbit | ab221552 | EPR20533-63 | 1/600 DVG | OPAL690 | 1/100 |
| PD-L1 | 4 | Rabbit | 64988 | D5V3B | 1/150 DVG | OPAL520 | 1/100 |
