# Supplementary material for: Sex differences in the aging murine urinary bladder and influence on the tumor immune microenvironment of a carcinogen-induced model of bladder cancer
Source: Biol Sex Differ. 2022 May 3;13:19. doi: 10.1186/s13293-022-00428-0 (PMC9066862; doi:10.1186/s13293-022-00428-0)
Supplement: Supplementary file 6 — Additional file 6. Detailed methods for RNA-Seq data analysis. [file 13293_2022_428_MOESM6_ESM.docx]

**Additional methods**

**RNA-sequencing and data analysis**

Libraries were prepared with a Ribo-zero rRNA depletion kit (Illumina) followed by sequencing using the illumina NovaSeq 6000 S4 PE100 platform. Quality of Fastq files was assessed using FastQC, and the presence of adapter sequences was observed. Reads were trimmed using Trimmomatic (0.36) using the following reference adapters; Forward: AGATCGGAAGAGCACACGTCTGAACTCCAGTCAC – Reverse: AGATCGGAAGAGCGTCGTGTAGGGAAAGAGTGT. Fastq files were then pseudoaligned against the mouse transcriptome (GRCm38.p6) from Gencode Release M25 using Salmon (1.3.0) in mapping mode with co-dependencies nixpkgs (16.09), openmpi (3.1.2), and gcc (7.3.0) on the Queen’s Centre for Advanced Computing cluster. This transcriptome is in ENSEMBL annotation. Raw and TPM normalized counts from salmon Quant.sf files were then extracted into R (4.1.0) using Tximport to get read coverage tables. TPM counts were visualized using Matlab R2020b for preprocessing. Differential expression of raw counts was performed then performed in R 4.1.0 using DESeq2. Genes were ranked by Log2foldchange for within sex age associated differences and between sex pairwise comparisons for all age groups.

Given that GSEA uses human gene sets, the ranked lists were collapsed and remapped using the Mouse_ENSEMBL_Gene_ID_Human_Orthologs_MSigDB.v7.4.chip before GSEA pre-ranked was run for 1000 permutations, with inclusion criteria for gene sets set from 15 to 200.^21^ Results were visualized through dot plots generated with ggplot2 (3.3.5) in R (1.4.0) representing the top 10 upregulated and downregulated GO Biological processes pathways in aged versus young female and male mice and age-matched females vs. males. Results of all significantly upregulated and downregulated pathways were also visualized using Cytoscape (3.8.2; http://www.cytoscape.org) based on the workflow developed by Reimand et al.^21^ Briefly, Enrichmentmap was run with FDR q-value < 0.05 and combined coefficient >0.375 with combined constant = 0.5 within Cytoscape. Nodes were clustered and labelled using AutoAnnotate run with the default MCL cluster algorithm with similarity coefficient edge weight column and a maximum of 10 annotations before being manually arranged and renamed to represent major biological themes. Given that genes were in ENSEMBL annotation in the TPM and raw data tables, genes from raw and TPM read tables with a known name were extracted and renamed using the org.Mm.eg.db package in R Bioconductor (3.13). DESeq2 was rerun using this raw data for all comparison groups and filtered with false discovery rate (FDR) adjusted *P-*value <0.05 (false discovery rate). Venn diagrams were generated using the VennDiagram packaged (1.6.2) in Rto reveal the overlaps between profiles of significantly overexpressed genes (*P*-adj <0.05, FDR with a log2foldchange cut off of 0.58) that associated with age and sex.
